# Cooks and Cooking

**Published:** 1889-11-02

**Authors:** 


### Cooks and Cooking.

The removal of the National Training School of Cookery to new and more commodious premises in Buckingham Palace-road may fairly be regarded as an event of some importance. We are getting to appreciate the value of cooking, and to feel no disgrace in the fact that men—and women too—must eat to live, and must eat well if they are to work hard and live happily. There is something modern about the notion. Though our fathers had "hearty" appetites, it was the fashion to look upon them as gross persons, to be regarded with a touch of refined contempt. As for ladies, of course, they had no appetites—at least, in public. Byron said he could not bear to see a woman eat, and the remark was held to be a sign of a sensitive, poetic nature. If *le Byron de nos jours* repeated the sentiment we should take him for a very affected, foolish fellow. Physiology has at least taught us that dyspepsia, however brought about, is a wrong to that body which God meant to be full of beauty and strength. Cooking has become an art, the teaching of cooking a recognised profession, and the existence, growth, and prosperity of a National Training School of Cookery is an admission of its status.

A list of names associated with the school shows at once what esteem it claims. The patron is the Prince of Wales, the president the Duke of Westminster, and the vice-president the Marquis of Northampton. Among the executive committee are numbered the Hon. E. F. Leveson-Gower, Sir Daniel Cooper, Sir Philip Cunliffe-Owen, Sir Edmund Henderson, and the Hon. Lyulph Stanley. Several of these are honoured names, though not as men who are high priests of *gourmandise*. The School of Cookery does not exist to permit those who lived daintily before to live more daintily still; but to spread through the length and breadth of the land a knowledge of the chemistry and hygiene of food, as well as of the most appetising and nutritious methods in which it can be prepared. And so it comes that among the examiners is Prof. Church, F.R.S.

"God sent meat, but the devil sent cooks," saith the proverb; and the elimination of diabolic interference with any divine gift is a thing to be devoutly desired. Moreover, at a time when the struggle for existence is terribly keen, when in so many households it is difficult to make both ends meet, and a housewife's constant endeavour is to make one shilling go as far as two, a knowledge of the art of making wholesome and appetising dishes out of cheap materials is a matter of no mean importance. Mistresses express themselves as shocked at the waste indulged in by young servants, who are extravagant through sheer ignorance. But meanwhile their own daughters grow up hardly knowing how to buy a joint, and certainly incapable of cooking it. Now with the first class, the servant girls, daughters of artisan fathers, when are they to learn cooking? They are compelled to go to school up to a certain age, after which it is generally necessary for them to go out and earn their living. All the cooking in their home has been done during their school hours, and they have seen none of it. Yet that they should know nothing of cooking is a serious matter for them when they go into service, and a still more serious matter when they marry and form homes of their own. If then the School of Cookery did nothing more than provide teachers who shall instruct the girls in our Board schools in the preparation of good and simple food, it would be an institution worthy of national support. In London, at least, these girls, numbering 20,000, are taught cooking, and it is to be hoped that in this all towns will follow the example of the metropolis, so that throughout the country the girls of the artisan class shall have a fundamental knowledge of the art of cookery.

This teaching of plain cookery, and training of teachers to give instruction in it, is one of the features of the school. But, as Sir Philip Cunliffe-Owen pointed out at the opening of the new premises, he who can afford to keep a cook is still a man and a brother, and deserves to have his comfort considered as much as the artisan. He perhaps wants more than his artisan brother, when he sends his cook to study the mysteries of *entrées* and his daughter to learn the art of pastry; and in the latter part the training differs; but for each a course of twenty lessons is required, and each course begins with practical instruction in scullery work. This scullery work is not pleasant, but it is fundamental. It is told of the late Mr. Buckmaster, a prophet among cooks, that being once honoured with a command to give a demonstration of the making of omelets before the Queen, he began by saying, "May it please your Majesty, if you want a good omelet you must first see that your frying-pan is clean." Good advice, this, for all cooks, from the Queen—of England, or of hearts—down to the humblest cottager's wife. Without cleanliness in every utensil employed, cooking becomes a thing unpleasing to the thought. But scullery training includes not merely the cleaning of pots and pans, but the best way of lighting and managing a fire, cleaning a fireplace, managing an oven, and other things, without which good cooking is impossible. Even if the cook does not do all these things herself, it is necessary that she should know how they should be done, or she cannot instruct her subordinate. The best prepared dish in the world may be spoiled in what the Scoth call the "firing," and ignorance of scullery work has as much as anything else to do with the sad results we have all experienced.

It must not be supposed that plain cookery means rough or coarse cookery. It is adapted to every middle-class household, and includes various methods of dealing with cold meat that would largely modify the bi-weekly monotony of hot, cold, and hash, which is so common in this favoured land. But it brings forward methods of cooking vegetables, cheap and wholesome soups—called maize because not founded on an expensive stock, but by no means lacking in nourishment—which would increase both the economy and the comfort of many households. We English have, perhaps, an exaggerated respect for a joint, and doubt if we can be fed without it. We know little of savoury stews, whilst of the daintier methods of preparing the cheaper kinds of fish we are sadly ignorant. Moreover, every doctor can tell, by sad experience, that a very large number of women are grievously ignorant of sick-room cookery. At the Training School this forms an essential part of the course, and some years ago teachers were sent down to Netley to give instruction to the nurses and orderlies there in that useful art.

As much economy as is compatible with satisfactory results, is the motto of the school; and as it proposed to open a restaurant in connection with it, it will be open to all the world to learn in the most practical fashion what the results are. The manner in which the money for the new abode of the school was raised is a proof that the two things are not incompatible. When the Fisheries Exhibition was opened, Mrs. Clarke, the lady superintendent of the National Training School, was aked to provide cheap fish dinners, in order, chiefly, to instruct the visitors to the exhibition in the good value of fish. These became so popular that new series were prepared for each successive exhibition. The profit of these sixpenny and shilling dinners amounted in the aggregate to £5,000, and with this the new building has been erected. With all our hearts we wish success to the National Training School of Cookery in its new home, and hope that the 40,000 pupils it has already sent out to illuminate our kitchens may prove but pioneers of an era of gastronomic peace and domestic content.

